# Recurrent Deep Vein Thrombosis (DVT) and Inferior Vena Cava (IVC) Filter: Should a Computed Tomography (CT) Venogram and Inferior Vena Cavagram Be the Standard of Care?

**DOI:** 10.7759/cureus.58529

**Published:** 2024-04-18

**Authors:** Kathleen Huynh, Anju Sanchala, Ayfer Ekiz

**Affiliations:** 1 Internal Medicine, Touro College of Osteopathic Medicine, Middletown, USA; 2 Physical Medicine and Rehabilitation, Garnet Health Medical Center, Middletown, USA; 3 Internal Medicine, Garnet Health Medical Center, Middletown, USA

**Keywords:** thromboembolism, ivc filter, pulmonary embolism, inferior vena cavagram, ct venogram, deep vein thrombosis

## Abstract

A pulmonary embolism (PE) is a life-threatening complication of deep vein thrombosis (DVT). Although timely anticoagulation is the first-line treatment for DVT, an inferior vena cava (IVC) filter can be considered when anticoagulation is contraindicated. Unfortunately, IVC filters come with complications of their own, including thrombus formation in or around the filter.

An 89-year-old man with a past medical history of coronary artery disease, congestive heart failure, chronic obstructive pulmonary disease, and prior DVT status post IVC filter implantation five years ago in 2018 presented with hypotension, dizziness, and syncope. Computed tomography angiography (CTA) of the chest showed bilateral PEs. Venous Doppler ultrasound of the bilateral lower extremities was negative for DVT. CT venogram was performed; however, the contrast filling was suboptimal and as such, a venous thrombosis could not be ruled out. Therefore, an inferior vena cavagram was performed through the right common femoral vein and confirmed a large thrombus positioned cephalad to the IVC filter. A thrombectomy was performed and the IVC filter was replaced given the patient was at high risk for venous thromboembolism recurrence and complications.

Although an IVC filter offers some protection from recurrent PEs, it does have risks and complications. As seen in our patient, the IVC filter can be a nidus for the formation of a thrombus which has the risk of dislodging. When evaluating a patient for the source of a PE, it is important to consider prior IVC implant and perform further workups, such as a CT venogram or an inferior vena cavagram, to evaluate for thrombus in or around the filter.

## Introduction

Pulmonary embolism (PE) is the most fatal complication arising from deep vein thrombosis (DVT), ranking as the third leading cardiovascular cause of death [1]. Following a diagnosis of DVT, timely initiation of anticoagulation is imperative [1]. However, in situations where anticoagulation is either contraindicated or ineffective, inferior vena cava (IVC) filters can be an alternative. An IVC filter functions as a physical trap for blood clots migrating from the lower extremity to the lungs, subsequently allowing dissolution through the body’s inherent thrombolytic mechanism, thus preventing the onset of pulmonary embolism [1]. 

The PREPIC (Prevention du Risque d'Embolie Pulmonaire par Interruption Cave) trial is a well-known trial that evaluated the efficacy of retrievable IVC filters [2]. The data showed the use of an IVC filter initially reduced the occurrence of PE. However, over time, the incidence of DVT was increased compared to those without filters [2]. To better understand the long-term complications of IVC filters, another study was conducted. This time, researchers performed an eight-year follow-up. It was again found that IVC filters reduced the risk of pulmonary embolism, however increased the risk of recurrent DVTs. Interestingly, of the 57 patients found to have recurrent DVT in the IVC filter group, 26 had a thrombus at the site of the IVC filter [3]. 

Following the PREPIC trial, IVC filter placement substantially increased, with an estimation of about a quarter of a million IVC filter placements per year [4]. While recurrent DVT is a well-known complication following IVC filter insertion, IVC thrombosis, specifically, occurs less frequently with incidence ranging from 0.6% to 18% [5]. Here, we present a case of bilateral PEs due to an atypical thrombus propagation across the apex of an IVC filter and discuss the challenges involved in its management. 

## Case presentation

An 89-year-old man (BMI 32.58 kg/m2) with a past medical history of coronary artery disease (post-3-vessel-coronary artery bypass), congestive heart failure, chronic obstructive pulmonary disease, hypertension, hyperlipidemia, and a previous DVT status post Greenfield^TM ^IVC filter (Boston Scientific Corp, MA, USA) placement in 2018 (anticoagulation was contraindicated in 2018 due to recurrent bleeding secondary to peptic ulcer disease), presented to the emergency department with hypotension, dizziness, and syncope. Upon admission, the patient denied experiencing shortness of breath, chest pain, leg swelling, or calf pain. Vital signs included a blood pressure of 80/50 mmHg, a heart rate of 50 beats/min, and an oxygen saturation of 98% on room air. Significant laboratory abnormalities were lactic acid of 4 mmol/L, potassium of 5.5 mEq/L, blood urea nitrogen (BUN) of 53 mg/dL, and creatinine of 1.97 mg/dL. A cardiac workup revealed sinus rhythm with first-degree atrioventricular block on electrocardiogram, along with negative troponin and normal B-type natriuretic peptide levels. Chest x-ray and computed tomography (CT) of the abdomen and pelvis with intravenous contrast were unremarkable. However, a CT angiography (CTA) of the chest revealed pulmonary emboli extending from branches to the left upper lobe and slightly protruding into the left pulmonary artery. Occlusion was noted in the left subclavian vein. There were smaller pulmonary emboli noted in the branches of the right upper lobe as well. The patient was administered crystalloid fluid boluses and sodium zirconium cyclosilicate, initiated on intravenous heparin, and transferred to the intensive care unit. 

**Figure 1:**
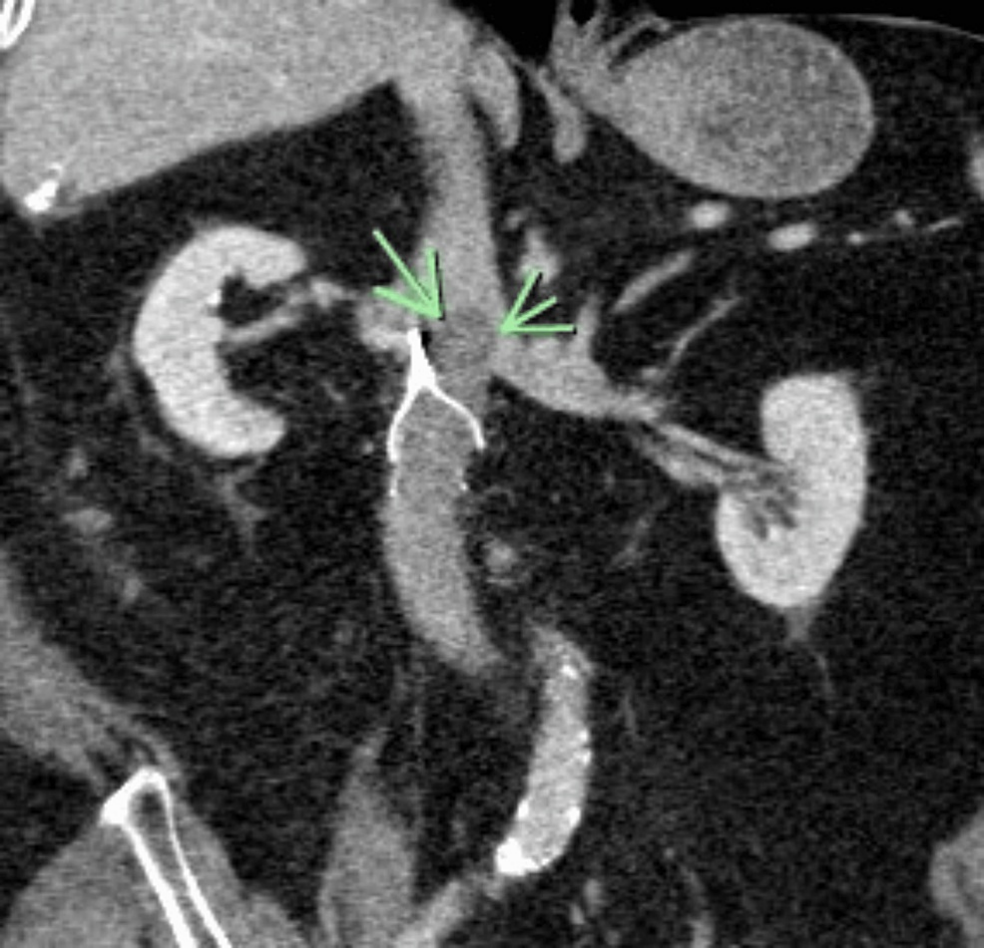
CT venography (the green arrows point to a possible thrombus positioned cephalad to the IVC filter on this coronal view). IVC: Inferior vena cava.

In an effort to determine the source of the embolism, a bilateral lower extremity venous doppler was done, which was negative for acute DVT. A 2D transthoracic echocardiogram showed low normal systolic function without evidence of McConnell’s sign or right ventricular strain. Given the lack of follow-up on the IVC filter placement over the past five years and to mitigate the risk of recurrent PE, a CT venogram of the abdomen and pelvis was performed but revealed suboptimal contrast filling of the veins and inconclusive results (Figure 1).

**Figure 2:**
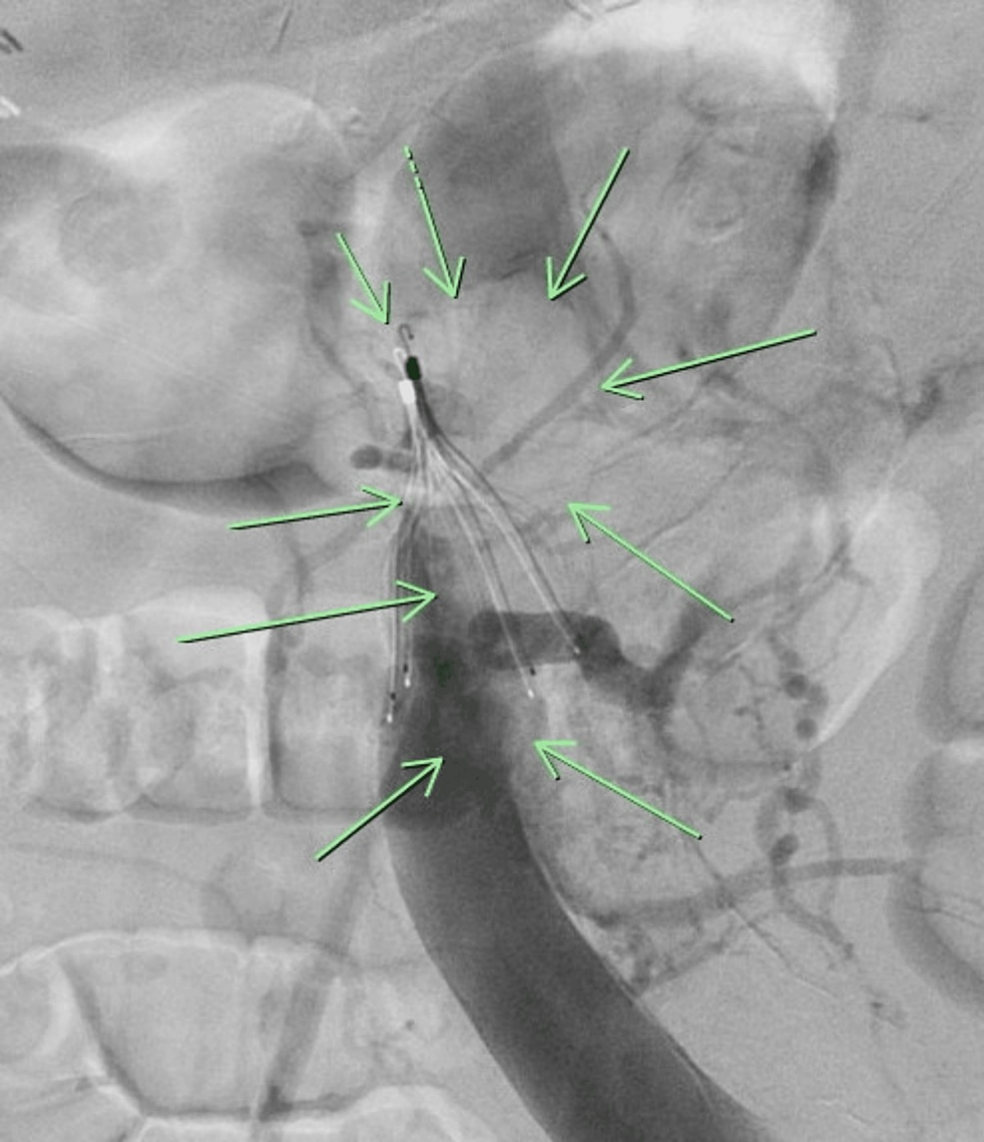
Inferior vena cavogram (the green arrows point to a large thrombus positioned cephalad to the IVC filter). IVC: Inferior vena cava.

The following day, an inferior vena cavagram through the right common femoral vein was performed revealing a large thrombus extending from the apex of the filter superiorly, partially protruding into the left renal vein, and a right-tilted IVC filter (Figure 2). Thrombectomy was performed through the right internal jugular vein, using a 20 French FlowTriever (Inari Medical, Irvine, CA, USA) suction embolectomy catheter. Multiple aspirations successfully targeted the thrombus, and a gooseneck snare facilitated the removal of the previously placed Greenfield^TM^ IVC filter. A new prophylactic Option^TM^ Elite IVC filter (Argon Medical Devices, TX, USA) was centrally deployed in the IVC at an infrarenal location. The patient tolerated the procedure well with no complications. Notably, there was a decrease in BUN/creatinine ratio from 33/1.72 mg/dL to 23/1.42 mg/dL after the procedure.

As part of the workup of the patient’s history of gastrointestinal (GI) bleed, an esophagogastroduodenoscopy and colonoscopy were performed, which showed no evidence of active upper and lower GI bleed, respectively. The patient’s symptoms and hemodynamic status improved, and he was discharged after a two-week hospitalization with clearance for long-term anticoagulation using rivaroxaban. Six months later, the patient’s condition is improving and he reports doing well without any complications since his discharge. He remains on rivaroxaban and has not experienced any shortness of breath, chest pain, or lower extremity discomfort.

## Discussion

Pulmonary embolism secondary to venous thromboembolism (VTE) is a preventable cause of death in hospitalized patients, with pharmacological anticoagulation being the first-line treatment. Classic indications for IVC filter placement as alternative management are when anticoagulation is contraindicated (uncontrollable active gastrointestinal bleeding or acute hemorrhagic stroke) or anticoagulation has failed [6]. Additionally, the Society of Interventional Radiology has proposed expanded indications that are relevant to our patient, including a massive PE treated with thrombectomy, ongoing VTE with limited cardiopulmonary reserve, and a recurrent PE despite having a filter in place, which collectively increases his risk for complications from VTE or anticoagulation [6]. In the United States, there are two main types of IVC filters utilized: permanent filters, which have been used since the 1970s, and retrievable filters, which were introduced in the late 1990s [6]. Permanent filters, such as the Greenfield^TM^ filter our patient initially presented with, are indicated for those who need long-term prophylaxis against PE, for those who cannot receive anticoagulation [6,7]. Retrievable filters, like the Option^TM^ Elite IVC filter which was later placed in our patient, have more flexibility in management as they have the option to be removed after the short-term risk of PE or contraindication to anticoagulation has been resolved [6,7]. Notably, the removal of retrievable filters can potentially lower the risk of long-term complications associated with permanent filters, including increased risk of future DVTs, filter migration or embolization, and IVC occlusion [6].

Due to the long-term increased risk of recurrent DVT, being able to remove an IVC filter is an attractive feature of the device. Unfortunately, as showcased in a systematic review, only 12-45% of patients have the device removed [8]. Additionally, the greatest number of device-related complications were found to occur after 30 days of IVC filter placement. The average time of filter retrieval was measured to be 72 days [8]. These numbers highlight an area of downfall regarding IVC filter use. More emphasis needs to be placed on follow-up after implantation of IVC filters. Of note, within the Society of Interventional Radiology Clinical Practice Guideline for IVC Filters in the Treatment of Patients with VTE Disease, it is suggested that a structured follow-up program be set in place to increase retrieval rates and detect complications at an early stage [9]. Specifically, there have been studies showing multidisciplinary teams, automated reminder systems, strong patient and physician education, and dedicated IVC filter clinics are helpful in decreasing complications due to prolonged IVC filter use [9]. Our patient originally had a permanent IVC filter implanted. Although retrieval of the filter was not planned, follow-up visits to monitor potential complications should have been scheduled. Upon speaking with the patient and his partner, they were unaware of any planned follow-up or any of the risks associated with long-term IVC filter use. 

Although IVC filters offer short-term PE protection, they come with various risks, highlighting the complexities involved in their management. One complication is IVC filter thrombosis, which was seen in our patient. This can result from emboli being trapped within the filter, the extension of DVT from lower extremities, or in situ thrombosis caused by the intrinsic thrombogenicity of the device [10]. The presentation of IVC thrombosis can vary widely, from no symptoms at all to severe lower extremity pain and edema [10]. While ultrasound with Doppler of the lower extremities is generally the first diagnostic approach, it can be limited when evaluating the IVC [10]. Clinicians must be diligent in obtaining a patient’s medical history and ensure that any existing IVC filter is evaluated with further diagnostic workup such as CT venography or inferior vena cavagram to evaluate for thromboembolic events in the deeper veins of the abdomen. This is particularly important in patients presenting with renal failure given the risk of thrombus propagation into the renal veins [10]. Current DVT algorithms lack IVC filter consideration even though IVC filter thrombus is a known complication. We propose integrating additional diagnostic workup for patients with an IVC filter into the standard DVT diagnosis algorithm which contains the Wells rule, d-dimer assay, and compression ultrasonography.

## Conclusions

Although inferior vena cava (IVC) filters offer some protection, they come with their own set of risks. As seen in our patient, the IVC filter is a location in which a thrombus can form, and thus also be dislodged. When evaluating a patient for the source of pulmonary embolism, it is important to consider prior IVC implantation and perform a CT venogram or inferior vena cavagram to evaluate for thrombus in and/or around the filter. Further research would need to be conducted regarding the cost-benefit analysis of this addition to the deep vein thrombosis workup for patients with any type of IVC filter.
